# Assessment of childhood undernutrition in India using National Family Health Surveys: Severity of anthropometric failure and contributing factors

**DOI:** 10.1371/journal.pone.0336335

**Published:** 2026-02-11

**Authors:** Ramendra Nath Kundu, Md. Golam Hossain, Susmita Bharati, Ummay Ayesha, Shahara Sultana Shanta, Premananda Bharati

**Affiliations:** 1 Department of Anthropology, West Bengal State University, Kolkata, West Bengal, India; 2 Department of Anthropology, Dr. Harisingh Gour Vishwavidyalaya (A Central University), Sagar, Madhya Pradesh, India; 3 Health Research Group, Department of Statistics, University of Rajshahi, Rajshahi, Bangladesh; 4 SEGi University, Selangor Darul Ehsan, Malaysia; 5 Sociological Research Unit, Indian Statistical Institute, Kolkata, West Bengal, India; 6 Department of Statistics, Comilla University, Comilla, Bangladesh; 7 Biological Anthropology Unit, Indian Statistical Institute, Kolkata, West Bengal, India; Christian Medical College, INDIA

## Abstract

**Background:**

Childhood undernutrition has a negative impact on healthy adulthood. Though progress is being made, a considerable number of children are still undernourished in India. The purpose of this study is to evaluate the prevalence of the severity of anthropometric failure (SAF) and its associated factors in India.

**Methods:**

This study was carried out with a cross-sectional design at the household level. The data we utilized were secondary in nature and collected from all five phases of the National Family Health Surveys from 1992 to 2021. This study comprised 581124 under-five children in India. The severity of anthropometric failure (SAF) was assessed using the composite index of anthropometric failure (CIAF). Children can experience SAF in four ways, categorized as no anthropometric failure (AF), single AF, double AF, or triple AF.

**Results:**

Over the past three decades, the prevalence of AF among under-five children in India has decreased. The latest NFHS survey indicates that the prevalence of AF was notably higher in rural areas (54.74%) compared to urban areas (47.73%). Single AF was a major issue in both rural (26.12%) and urban (25.37%) areas, while double AF presents a greater concern in rural areas (23.36%). Several socio-demographic and maternal factors have been identified as significant contributors to AF, particularly concerning low birth weight (LBW) and poor wealth index in both urban and rural contexts. In urban settings, AF was more prevalent among Muslim children and those whose mothers had lower levels of education. In rural areas, the condition was more common among children of underweight mothers and those from scheduled castes and scheduled tribes.

**Conclusions:**

More than two-fifths of under-five children have either S-AF or D-AF, which was significant. The notably higher rates of undernutrition in rural areas highlight the urgent need for targeted interventions. Addressing these issues requires a comprehensive approach that focuses on improving maternal health, increasing educational opportunities, and implementing community-based nutrition programs, especially for vulnerable groups such as those from scheduled castes and tribes.

## Introduction

Deficiencies at an early age are not irreversible, especially in under-five children. Childhood undernutrition is a problem that can lead to various health problems throughout life. Proper nutrition in early childhood is critical for organ development and function, a strong immune system, and neurological and cognitive improvement [1]. UNICEF, WHO, and World Bank Group estimated that in 2022, there were 148.1 million stunted (low height-for-age) and 45 million wasted (low weight-for-height) children worldwide [2]. The WHO reported that stunting had decreased worldwide in 2021, except for Africa. Asia is home to more than three-quarters of children affected by severe wasting and more than half (25 million) of all children who suffer from wasting live in the southern part of Asia [3].

According to the WHO, in 2021, undernutrition is responsible for 45 percent of deaths among under-five children, predominantly in low- and middle-income countries (LMICs) [4]. At the same time, undernutrition is identified by UNICEF (2020) as the prime cause of over half of all fatalities in under-five children [5]. Child undernutrition has numerous underlying causes. Previous studies in several nations have identified multiple factors contributing to undernutrition, including inadequate supplemental nutrition, poor socioeconomic characteristics, poverty, co-morbidities, and food insecurity [6–8]. These factors are linked to the nation’s economic and healthcare systems, which change over time and have an impact on the prevalence of child undernutrition [9–11].

Anthropometry helps to determine childhood undernutrition [12,13]. The three most common anthropometric indicators for undernutrition in children are low height-for-age or stunting, weight-for-height or wasting, and weight-for-age or underweight [14,15]. A child may exhibit various forms of undernutrition at once, such as stunting and underweight or wasting and underweight [16]. All three forms can be observed together in the same child occasionally. The Composite Index of Anthropometric Failure (CIAF) provides a comprehensive framework for understanding childhood undernutrition by integrating all these undernutritional indicators into seven distinct subgroups [6,7]. This concept was initially introduced by Svedberg in 2000, focusing on children who may exhibit signs of stunting, wasting, or underweight—critical indicators of anthropometric failure [6,16]. This initial CIAF model comprised six subgroups, labelled A through F [16]. In 2005, Nandi et al. expanded this model by utilizing data from India [17]. This adaptation included the introduction of an additional subgroup, labelled Y, enabling researchers and policymakers to assess childhood undernutrition more effectively [6–8,17].

Previous research indicates that factors such as limited wealth, lower social status, insufficient maternal education, and maternal undernutrition can contribute to issues like childhood stunting, wasting, and underweight [13,14,18]. In this regard, we can more effectively identify the key determinants by evaluating the overall magnitude of AF, rather than considering the CIAF subgroups separately. This approach will enable LMICs to develop more cost-effective strategies for addressing childhood undernutrition and achieving the Sustainable Development Goals (SDGs). The magnitude and vulnerability of childhood undernutrition are the foundation of the current investigation because of this. The objective of this study is to assess the prevalence and severity of anthropometric failure (SAF), identify socio-demographic and maternal characteristics in both urban and rural settings, and examine its trends over the past three decades.

## Methods

### Conceptual framework

**Fig 1:**
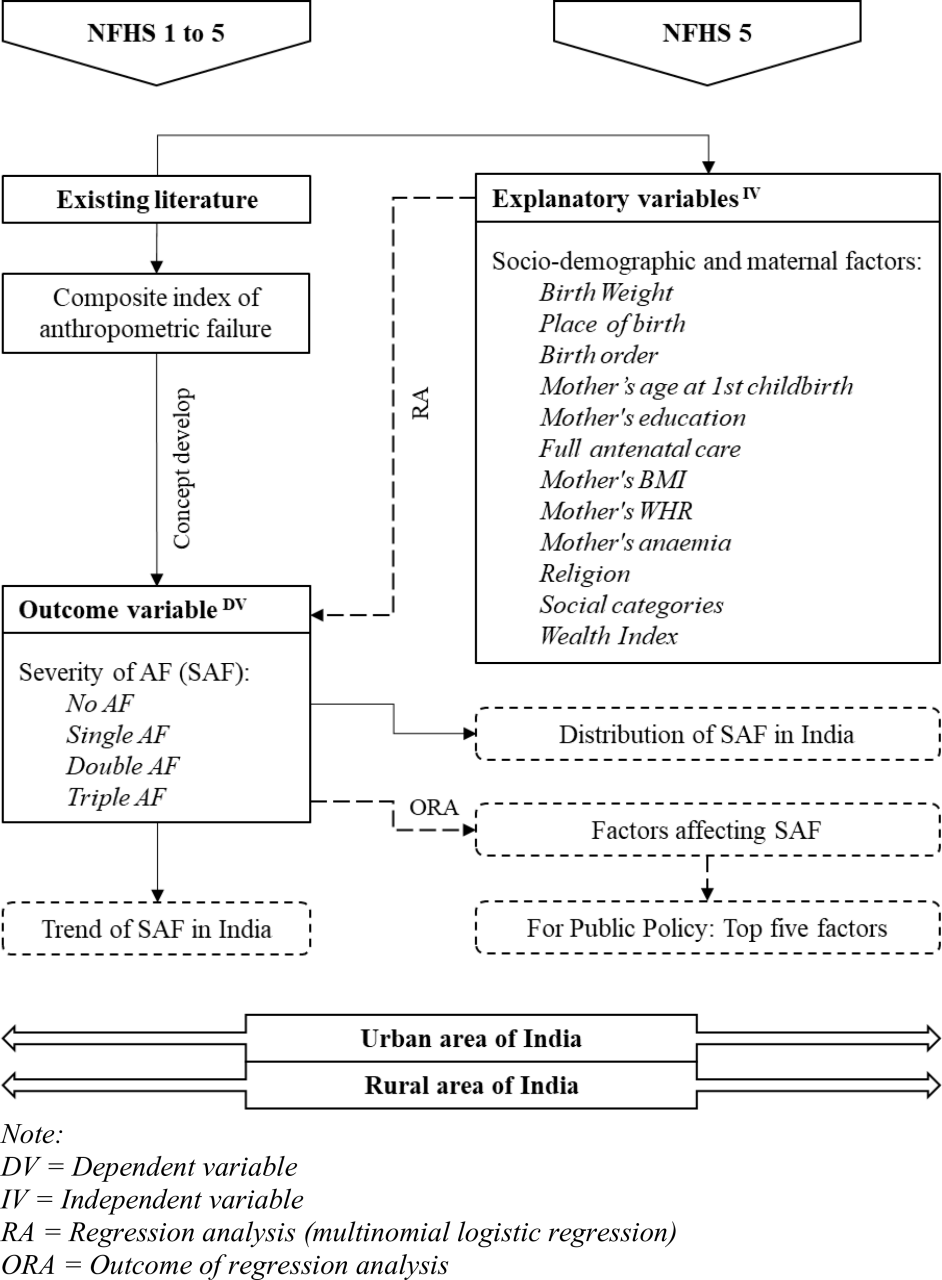
Conceptual framework of the study.

The study’s purpose is to investigate the severity of anthropogenic failure (SAF) in urban and rural India from both prospective and cross-sectional perspectives (Fig 1). The concept of SAF was developed based on the existing concept of CIAF from the literature. The study analyzes the trends in SAF based on data from the National Family Health Survey (NFHS) spanning between 1992−93 (NFHS 1) and 2019−21 (NFHS 5) to understand the changes in SAF over time. The latest distribution of SAF in India is established based on NFHS 5 data. NFHS-5 fieldwork for India was conducted in two phases— Phase-I from 17 June 2019–30 January 2020 covering 17 states and 5 UTs and Phase-II from 2 January 2020–30 April 2021 covering 11 states and 3 UTs. The study also identifies the socio-demographic and maternal factors influencing SAF based on recent data.

### Data source

The unit-level data used in this study were collected from five phases of the NFHS, which include NFHS 1 (1992−93), NFHS 2 (1998−99), NFHS 3 (2005−06), NFHS 4 (2015−16), and NFHS 5 (2019−21). This cross-sectional survey was conducted in households across the country between 1992 and 2021 [19]. A total of 581124 under-five children were selected from 620041 women of reproductive age; out of these selected children, 533478 were selected for statistical analysis of CIAF.

### Inclusion and exclusion criteria

**Fig 2:**
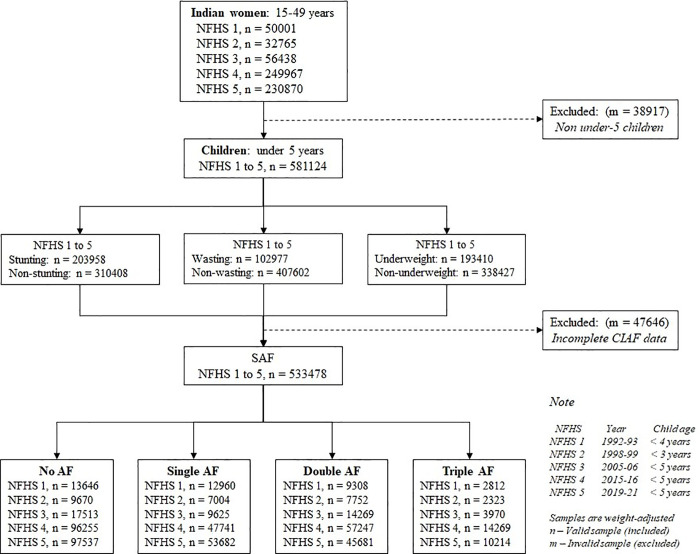
Sample selection for SAF of under-five children.

The only Indian women included in this study were of reproductive age (15–49 years) and living in India. Children under five years of age were considered among the selected mothers for this study. The sample selection strategy for the study was illustrated in the following flow chart (Fig 2).

### Patient and public involvement

This study solely relies on secondary data derived from the NFHS in India. During data collection, proper consent was obtained from the participants following the guidelines of the Demographic and Health Survey (DHS). This study has properly utilized the patient and public involvement (PPI) to upgrade the research quality. Although the public was indirectly involved in this study, the study’s outcomes may directly benefit the public domain. As the NFHS data adheres to the standard methodology, participant privacy, and human subjects’ protection, policymakers rely on this information to take appropriate steps to enhance the public health policies that ultimately benefit the citizens of the country.

### Unit-level study variables

#### Outcome variables.

The outcome variable for this study was the SAF, which was developed based on the established CIAF classification (S1 Table). The description of SAF is thoroughly mentioned in the Operational Definition and Classifications section. According to the literature, the current CIAF classification consists of seven subgroups [20,21]. The first subgroup (Group A) is designated as No-Anthropometric Failure (N-AF) because it does not include any undernutrition conditions. In contrast, the remaining subgroups (Groups B to Y) encompass various undernutrition conditions, collectively referred to as anthropogenic failures (AF) [20,21]. The AF signifies inadequate or poor nutrition, assessed through nutritional indicators such as wasting, stunting, and underweight [21]. These indicators were determined in accordance with WHO recommendations, which defined a z-score of less than –2.0 SD for weight-for-height as wasting, height-for-age as stunting, and weight-for-age as underweight [22].

#### Explanatory variables.

Socio-demographic and maternal characteristics were included in the explanatory factors. Explanatory factors were selected and categorized based on previous studies while retaining their meaning and were available in the NFHS-5 dataset. Birth weight (LBW, non-LBW), place of birth (home, institution), birth order (≤2, > 2), mother’s age at 1st childbirth (<18, 18–25, > 25), religion (Hindu, Muslim, Christian, Other), social categories (scheduled tribe, scheduled caste, other backward classes, general), wealth index (poor, middle, rich) have been shown as socio-demographic factors [11,14,23]. Mother’s education (non-education, primary, secondary, higher), full antenatal care (yes, no), mother’s BMI (underweight, normal, overweight/obese), mother’s WHR (RMC, no-RMC), mother’s anaemia (anaemic, non-anaemic) have been shown as maternal factors [11,14,23].

### Operational definition and classifications

#### Severity of anthropometric failure (SAF).

Anthropometric failure (AF) was defined as children with wasting, stunting, and underweight, indicating insufficient height and weight for their age [24]. Children can exhibit these undernutrition symptoms in four different ways: absence of all undernutrition indicators, presence of any one indicator, presence of two indicators, presence of all three malnutrition indicators. Based on this occurrence, CIAF prevalence can be classified into four categories based on severity, namely no AF (N-AF), single AF (S-AF), double AF (D-AF), and triple AF (T-AF), as shown in S1 Table.

#### Socio-demographic and maternal factors.

Low birth weight (LBW) refers to birth weights <2500 grams, while non-LBW refers to birth weights ≥2500 grams [25]. The religion group “other” comprises Sikh, Buddhist/Neo-Buddhist, Jain, Jewish, Parsi/Zoroastrian, no religion, and other, with a total of less than 5%. The social category was defined by whether the head of the household self-identifies as a scheduled tribe (ST), scheduled caste (SC), other backward classes (OBC) member, the remaining caste/tribe population was determined as ‘general’ category [26]. Scheduled Castes, Scheduled Tribes, and Other Backward Classes are constitutionally recognized and protected communities in India. Scheduled Castes (SC) are those who have historically faced oppression, economic disadvantage, lack of education, political powerlessness, and cultural subordination to the upper caste. They are recognized under Articles 341 and 366 (24) of the Constitution. Scheduled Tribes are those who share a common culture, language, and territory, and who experience economic disadvantage. They are recognized under Articles 342 and 366 (25) of the Constitution. Other Backward Classes are economically disadvantaged and lack access to education, affecting their overall quality of life, including access to food, education, and healthcare. The wealth index measured a household’s wealth based on the quantity and variety of consumer goods it owns, its assets, and housing characteristics, which cumulatively weighted and splatted, with the richest families receiving the highest score and the poorest receiving the lowest [19]. Maternal education solely relates to formal institutional education. Full antenatal care was defined as ensuring four or more antenatal visits, at least one tetanus toxoid (TT) injection, and consumption of iron folic acid (IFA) tablets or syrup for at least 100 days [27]. Body mass index (BMI) was determined by dividing weight in kilograms by height in meters squared (kg/m^2^), and the obtained values were classified as underweight if it was < 18.5; normal was considered to be between ≥18 and <25; and overweight/obese was ≥ 25 based on WHO (2004) classification for adults [28]. According to the WHO, the Waist–Hip Ratio (WHR) for women indicates the risk of Metabolic Complications (RMC) [29]. A WHR value of ≥0.85 cm was classified as RMC, while a WHR value of <0.85 cm indicates no-RMC [29]. A woman was considered anaemic when her haemoglobin level was < 11 level in grams/decilitre (g/dl) [19].

### Statistical analysis

Summary of outcome and explanatory variables were defined descriptively between urban and rural India using frequencies and percentages. The Z-proportion test was used to assess the variations in proportional frequency between urban and rural areas. After performing a multicollinearity test, explanatory variables were chosen, with a variance inflation factor (VIF) of less than 5 being taken into consideration. The adjusted relative risk ratio (ARRR) was utilized to determine the combined impact of socio-demographic and maternal characteristics on SAF using multinomial logistic regression (MLR), where N-AF considered as reference in dependent variable in respect of S-AF, D-AF, and T-AF [30]. Statistical significance was considered based on 95% confidence interval (CI), and p ≤ 0.05. Statistical analyses were carried out using MS Excel and STATA.

### Ethics approval and consent to participate

The present study relies on nationally representative data from the National Family Health Survey 2019–2021 (NFHS-5) obtained from the Demographic and Health Survey (DHS) and available in the public domain. NFHS-5 obtained written consent from each selected subject. The ICF Institutional Review Board (IRB) examined and approved the study design and participant confidentiality, the NFHS-5 data were already ethically approved; therefore use of these data no longer required any other ethical approval. NFHS-5 discussed about the objectives of the survey to selected participants and obtained their written consent.

## Results

**Fig 3:**
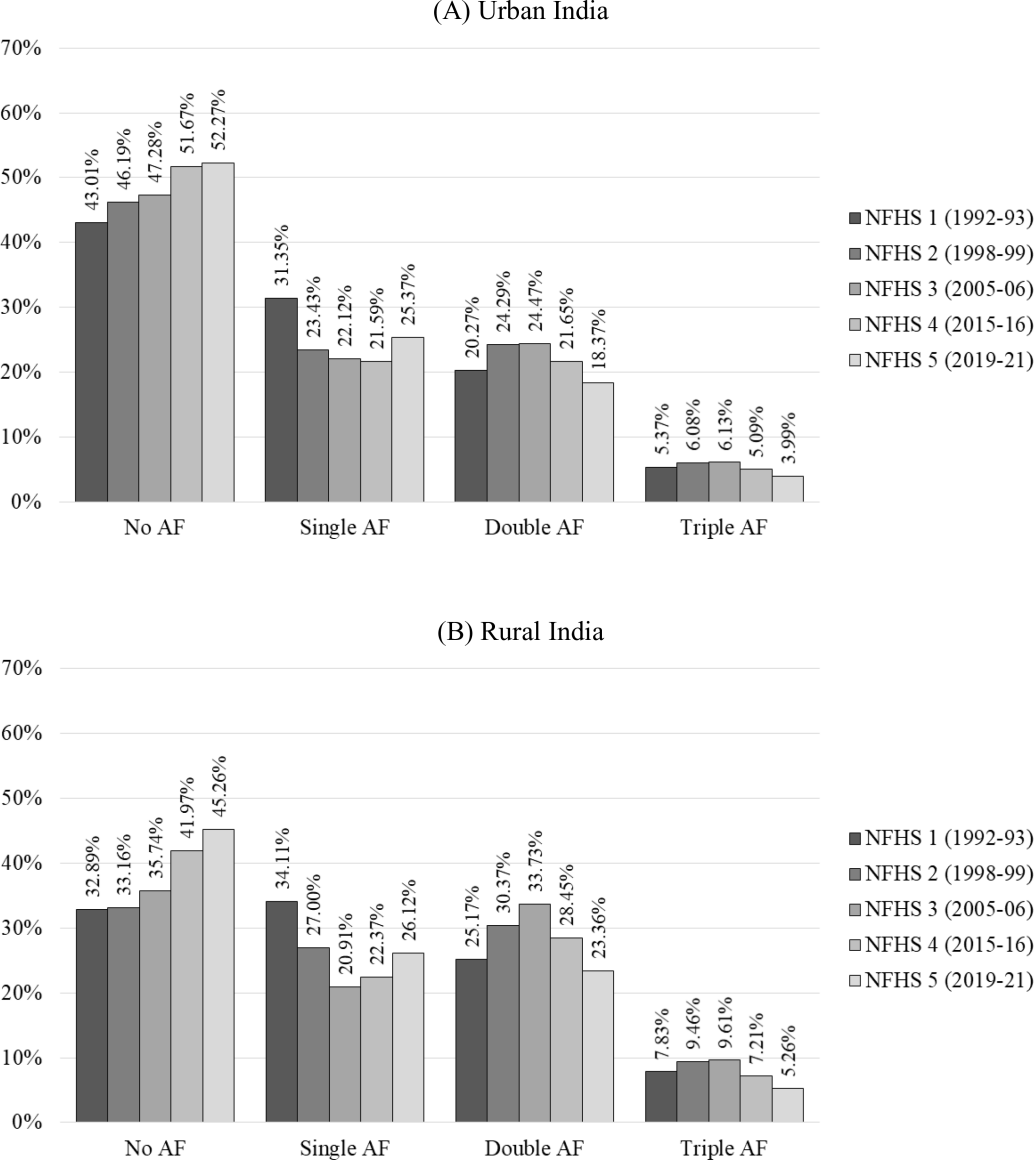
Decadal trend of SAF in Indian under-five children spanning between 1992 and 2021. **(A)** Urban India, **(B)** Rural India.

The National Family Health Survey (NFHS) data provides a comprehensive view of India’s progress in improving children’s health over the past three decades (Fig 3). The data indicate that the prevalence of N-AF has substantially increased in urban and rural children, and AF has declined.

Single AF (S-AF) is the most common form of anthropometric failure in India, affecting about one in three children according to NFHS 1 data. However, recent data from NFHS 5 shows improvement, with about one in four children affected—25.37% in urban areas and 26.12% in rural areas. Double AF (D-AF) and Triple AF (T-AF) were more common in rural children than urban children. The highest rates of D-AF were recorded in NFHS 3, where about one in four urban and one in three rural children were affected. By NFHS 5, these figures improved to one in five for urban and one in four for rural children. T-AF has also seen a significant drop, especially in recent data. In urban areas, it decreased from 6.13% in NFHS 3 to 3.99% in NFHS 5, while in rural areas, it dropped from 9.61% to 5.26%. Overall, S-AF remains a concern but reflects progress in addressing childhood undernutrition in India (Fig 3).

The latest NFHS 5 data shows that among urban Indian children, the highest prevalence of N-AF was found in Manipur (69.29%), Sikkim and Mizoram (68.75%), Chandigarh (67.72%). whereas in rural areas, Puducherry (67.50%), Punjab (66.24%), Delhi (65.59%) (S1 Fig). The highest prevalence of S-AF was observed in urban Lakshadweep (33.33%), Jammu & Kashmir (32.15%), Tripura (31.54%), whereas in rural Lakshadweep (50.00%), Ladakh (35.00%), Meghalaya (33.52%). The highest prevalence of D-AF was noted in urban Bihar (24.61%), Gujarat (22.38%), Dadra & Nagar Haveli and Daman & Diu (21.95%), whereas in rural Chandigarh (50.00%), Bihar (29.88%), Jharkhand (28.85%). The highest occurrence of T-AF was found in the states of Dadra & Nagar Haveli and Daman & Diu for both urban (7.32%) and rural (9.52%) areas, followed by Maharashtra (5.79%) and Bihar (5.72%) for urban, Gujarat (8.62%) and Maharashtra (7.78%) for rural area (S1 Fig).

Table 1 shows the differences in percentages between urban and rural areas using z-scores. A positive z-score means a higher percentage in urban areas, while a negative z-score means a higher percentage in rural areas. The data reveals that the percentages of N-AF, D-AF, and T-AF were significantly different between the two areas, with z-scores of 19.91 (p < 0.001), −10.59 (p < 0.001), and −2.41 (p < 0.05), respectively.

**Table 1:** Differences of proportion in variable categories between urban and rural areas based on the latest data (NFHS 5).

Unit-level Variables	Urban		Rural			
	n	Proportional % (95% CI)	n	Proportional % (95% CI)	Proportional z-score	p-value
***Outcome variable***						
Severity of AF						
No AF	28351	52.27 (51.85, 52.69)	69185	45.26 (45.01, 45.51)	19.91	<0.001
Single AF	13759	25.37 (25.00, 25.73)	39923	26.12 (25.90, 26.34)	−1.73	0.084
Double AF	9965	18.37 (18.05, 18.70)	35716	23.36 (23.15, 23.58)	−10.59	<0.001
Triple AF	2167	3.99 (3.83, 4.16)	8047	5.26 (5.15, 5.38)	−2.41	0.016
***Explanatory variables***						
Birth Weight						
LBW	10047	17.36 (17.05, 17.67)	28120	18.58 (18.38, 18.78)	−2.72	0.007
Non-LBW	47825	82.64 (82.33, 82.95)	123231	81.42 (81.22, 81.62)	5.86	<0.001
Place of birth						
Home	3693	6.01 (5.83, 6.21)	22155	13.11 (12.95, 13.27)	−12.25	<0.001
Institution	57709	93.99 (93.79, 94.17)	146828	86.89 (86.73, 87.05)	45.99	<0.001
Birth order						
≤ 2	48771	79.27 (78.94, 79.59)	119144	70.36 (70.15, 70.57)	37.31	<0.001
> 2	12757	20.73 (20.41, 21.06)	50198	29.64 (29.43, 29.86)	−20.05	<0.001
Mother’s age at 1^st^ childbirth						
< 18	5091	8.27 (8.06, 8.49)	21710	12.82 (12.66, 12.98)	102.19	<0.001
18-25	44660	72.59 (72.23, 72.94)	132360	78.16 (77.96, 78.36)	−24.10	<0.001
> 25	11777	19.14 (18.83, 19.45)	15272	9.02 (8.88, 9.16)	−117.93	<0.001
Mother’s education						
No education	6998	11.37 (11.13, 11.63)	42308	24.98 (24.78, 25.19)	−25.04	<0.001
Primary	5760	9.36 (9.13, 9.59)	22675	13.39 (13.23, 13.55)	−8.24	<0.001
Secondary	31515	51.22 (50.83, 51.62)	85516	50.50 (50.26, 50.74)	2.19	<0.001
Higher	17255	28.04 (27.69, 28.40)	18844	11.13 (10.98, 11.28)	40.74	<0.001
Full antenatal care						
Yes	30059	60.92 (60.49, 61.35)	60940	48.52 (48.24, 48.80)	35.23	<0.001
No	19281	39.08 (38.65, 39.51)	64658	51.48 (51.20, 51.76)	−30.23	<0.001
Mother’s BMI						
Underweight	7188	12.38 (12.12, 12.65)	36146	21.93 (21.73, 22.13)	−18.37	<0.001
Normal	33329	57.41 (57.01, 57.81)	103720	62.91 (62.68, 63.15)	−17.96	<0.001
Overweight/Obese	17538	30.21 (29.84, 30.58)	24992	15.16 (14.99, 15.33)	37.27	<0.001
Mother’s WHR						
RMC	35606	61.41 (60.96, 61.86)	92161	55.94 (55.71, 56.17)	17.73	<0.001
No RMC	22376	38.59 (38.14, 39.04)	72598	44.06 (43.83, 44.29)	−14.46	<0.001
Mother’s anaemia						
Anaemic	31513	55.14 (54.73, 55.55)	100576	61.65 (61.41, 61.88)	−20.59	<0.001
Non anaemic	25640	44.86 (44.45, 45.27)	62568	38.35 (38.12, 38.59)	17.90	<0.001
Religion						
Hindu	45176	73.42 (73.07, 73.77)	138162	81.59 (81.40, 81.77)	−37.39	<0.001
Muslim	13543	22.01 (21.68, 22.34)	23953	14.14 (13.98, 14.31)	19.50	<0.001
Christian	1359	2.21 (2.10, 2.33)	3425	2.02 (1.96, 2.09)	0.42	0.674
Other	1451	2.36 (2.24, 2.48)	3802	2.25 (2.18, 2.32)	0.24	0.810
Social categories						
Scheduled tribe	2616	4.52 (4.36, 4.70)	20524	12.78 (12.62, 12.95)	−12.31	<0.001
Scheduled caste	12407	21.46 (21.12, 21.79)	41350	25.75 (25.54, 25.96)	−9.71	<0.001
Other Backward Classes	26812	46.37 (45.96, 46.78)	73596	45.83 (45.59, 46.07)	1.52	0.129
General	15986	27.65 (27.28, 28.01)	25111	15.64 (15.46, 15.82)	29.50	<0.001
Wealth Index						
Poor	8310	13.51 (13.24, 13.78)	98631	58.24 (58.01, 58.48)	−78.68	<0.001
Middle	10695	17.38 (17.09, 17.68)	34406	20.32 (20.13, 20.51)	−6.69	<0.001
Rich	42523	69.11 (68.75, 69.48)	36305	21.44 (21.24, 21.64)	133.64	<0.001

A notable difference revealed in certain health and social factors between urban and rural areas. In rural areas, the prevalence of LBW was higher at 18.58% compared to 17.36% in urban areas. Home births were also more common in rural areas (13.11%) than in urban areas (6.01%). Birth order varied significantly, with urban areas seeing 79.27% of families having up to 2 children, while rural areas had a higher percentage (29.64%) of families with more than 2 children. Early childbirth among mothers under 18 was more prevalent in rural areas (12.82%) than in urban areas (8.27%). Additionally, over 70% of first-child births in both regions occurred between the ages of 18 and 25 years. Rural mothers showed higher rates of non-education (24.98%), incomplete antenatal care (51.48%), underweight BMI (21.93%), and anaemia (61.65%). In contrast, urban mothers had higher education levels (28.04%), full ANC (60.92%), overweight/obese BMI (30.21%), and risk of metabolic complications (RMC, 61.41%). Religiously, a majority of Hindu mothers resided in rural areas (81.59%), while Muslim and Christian mothers were more often found in urban regions. Socially, SCs and STs were more prevalent in rural areas (25.75% and 12.78%, respectively). Wealth index showed that rural areas had more poor and middle-class families (58.24% and 20.32%, respectively) compared to urban areas, which had a higher proportion of wealthy families (69.11%) (Table 1).

The data in Table 2 highlights how various socio-demographic and maternal factors influence the occurrence of different types of AF outcomes in urban India. All kinds of AF occur in LBW children, the prevalence of T-AF outcomes was 2.26 times (ARRR 2.26; CI 2.00, 2.56) higher in LBW children compared to non-LBW children. Children born at home have 1.45 times (ARRR 1.45; CI 1.11, 1.89) times higher likelihood of T-AF than those born in institutions. D-AF and T-AF outcomes were more prevalent in third or later births, being 1.38 and 1.19 times (ARRR 1.38, CI 1.28, 1.48; ARRR 1.19, CI 1.03, 1.36) more common, respectively. Pregnancies occurring between the ages of 18 and 25 show 1.22 times (ARRR 1.22; CI 1.04, 1.45) higher risk of T-AF compared to pregnancies over 25. Low maternal education significantly increases the risk of all types of AF, with the risk being highest 2.19 times (ARRR 2.19; CI 1.75, 2.73) for T-AF among mothers with only a primary education. Inadequate antenatal care contributes to 1.18 times (ARRR 1.18; CI 1.06, 1.32) higher risk of T-AF. Maternal underweight was associated with higher prevalence rates of all AF types, particularly T-AF (ARRR 1.61, CI 1.39, 1.87), while overweight/obesity in mothers shows a reduced risk across categories. Maternal anaemia also raises the risk of D-AF by 1.06 times (ARRR 1.06; CI 1.00, 1.13). Muslim children face a 1.29 times higher risk of S-AF and a 1.20 times higher risk of D-AF (ARRR 1.29, CI 1.21, 1.38; ARRR 1.20, CI 1.11, 1.30, respectively). Children from STs have a higher risk of S-AF and D-AF (ARRR 1.31, CI 1.16, 1.49; ARRR 1.20, CI 1.03, 1.38, respectively), whereas children of OBCs show 1.19 times (ARRR 1.19, CI 1.03, 1.36) higher risk for T-AF. Children from families with poor wealth index were more likely to experience all forms of AF, with T-AF prevalence being 1.67 times (ARRR 1.67, CI 1.41, 1.96), higher in poor families compared to rich families (Table 2).

**Table 2:** Factors affecting SAF in urban areas of India based on the latest data (NFHS 5).

Explanatory Factors	Single AF			Double AF			Triple AF		
	ARRR (95% CI)	z	p-value	ARRR (95% CI)	z	p-value	ARRR (95% CI)	z	p-value
Birth Weight (Non-LBW ®)									
LBW	1.33 (1.24, 1.42)	8.34	<0.001	1.84 (1.71, 1.98)	16.76	<0.001	2.26 (2.00, 2.56)	12.84	<0.001
Place of birth (Institution ®)									
Home	1.14 (0.98, 1.33)	1.73	0.084	1.03 (0.87, 1.22)	0.38	0.705	1.45 (1.11, 1.89)	2.75	0.006
Birth order number (≤ 2 ®)									
> 2	1.07 (1.00, 1.14)	1.86	0.063	1.38 (1.28, 1.48)	8.64	<0.001	1.19 (1.03, 1.36)	2.45	0.014
Mother’s age at 1st childbirth (>25 years ®)									
< 18 years	0.96 (0.85, 1.07)	−0.75	0.453	0.91 (0.80, 1.03)	−1.43	0.153	1.24 (0.97, 1.58)	1.71	0.087
18–25 years	1.02 (0.95, 1.08)	0.48	0.632	0.95 (0.88, 1.02)	−1.35	0.177	1.22 (1.04, 1.45)	2.38	0.018
Mother’s education (Higher ®)									
No education	1.26 (1.13, 1.40)	4.16	<0.001	1.71 (1.52, 1.92)	8.92	<0.001	1.88 (1.49, 2.37)	5.31	<0.001
Primary	1.20 (1.08, 1.33)	3.40	0.001	1.58 (1.40, 1.77)	7.66	<0.001	2.19 (1.75, 2.73)	6.93	<0.001
Secondary	1.17 (1.10, 1.24)	5.15	<0.001	1.37 (1.27, 1.48)	8.39	<0.001	1.78 (1.53, 2.09)	7.25	<0.001
Full antenatal care (Yes ®)									
No	0.98 (0.93, 1.03)	−0.77	0.440	1.02 (0.96, 1.08)	0.61	0.544	1.18 (1.06, 1.32)	2.99	0.003
Mother’s BMI (Normal ®)									
Underweight	1.13 (1.04, 1.23)	2.97	0.003	1.46 (1.34, 1.58)	8.73	<0.001	1.61 (1.39, 1.87)	6.41	<0.001
Overweight/obese	0.69 (0.65, 0.73)	−13.33	<0.001	0.56 (0.52, 0.60)	−16.65	<0.001	0.50 (0.44, 0.58)	−9.50	<0.001
Mother’s WHR (No RMC ®)									
RMC	1.04 (0.99, 1.09)	1.53	0.126	1.00 (0.94, 1.06)	−0.14	0.887	0.97 (0.87, 1.09)	−0.46	0.647
Mother’s anaemia (Non-anaemic ®)									
Anaemic	0.96 (0.92, 1.01)	−1.45	0.148	1.06 (1.00, 1.13)	2.09	0.036	1.01 (0.91, 1.13)	0.22	0.824
Religion (Hindu ®)									
Muslim	1.29 (1.21, 1.38)	7.51	<0.001	1.20 (1.11, 1.30)	4.69	<0.001	0.98 (0.84, 1.14)	−0.26	0.792
Christian	0.92 (0.78, 1.08)	−1.03	0.302	0.92 (0.76, 1.12)	−0.83	0.406	0.66 (0.41, 1.05)	−1.76	0.079
Other	1.25 (1.06, 1.46)	2.69	0.007	1.22 (1.01, 1.47)	2.03	0.042	1.65 (1.19, 2.29)	2.99	0.003
Social categories (General ®)									
Scheduled tribe	1.31 (1.16, 1.49)	4.21	<0.001	1.20 (1.03, 1.38)	2.40	0.017	1.15 (0.87, 1.53)	1.00	0.318
Scheduled caste	1.10 (1.02, 1.18)	2.54	0.011	1.17 (1.07, 1.27)	3.62	<0.001	1.13 (0.96, 1.33)	1.42	0.154
Other Backward Classes	1.02 (0.96, 1.08)	0.72	0.469	1.05 (0.98, 1.13)	1.42	0.156	1.19 (1.03, 1.36)	2.45	0.014
Wealth Index (Rich ®)									
Poor	1.13 (1.04, 1.24)	2.75	0.006	1.63 (1.48, 1.78)	10.42	<0.001	1.67 (1.41, 1.96)	6.11	<0.001
Middle	1.05 (0.98, 1.13)	1.46	0.145	1.25 (1.15, 1.35)	5.53	<0.001	1.36 (1.18, 1.57)	4.23	<0.001

**Note:**

• The table aims to identify the factors contributing to the severity of anthropometric failure (AF) in urban areas of India. To achieve this, the researchers used a Multinomial logistic regression (MLR) model because the dependent variable SAF was categorical and had four categories (SAF = No AF, Single AF, Double AF, Triple AF). In the model, “No AF” was used as the reference for the dependent variable, while “®” was used for independent variables.

• The Adjusted Relative Risk Ratio (ARRR) was used to indicate the cumulative odds of the independent factors for each category of the dependent variable relative to “No AF”. The 95% level of confidence interval was used to determine the lower and upper bounds of ARRR. Additionally, the z-value was used to indicate the rate of influence of the independent factors on each category of the dependent variables, while the p-value was used to indicate the significance level for ARRR.

• The study found that low birth weight, mother’s education, mother’s BMI, and poor wealth index are factors that are common to single, double, and triple AF.

In rural India, similar trends to urban areas were observed regarding the impact of socio-demographic and maternal factors on SAF. LBW children faced a higher risk for all forms of AF, with 2.33 times (ARRR 2.33, CI 2.18, 2.50) higher prevalence in T-AF. Home-born children were also more vulnerable, exhibiting risks 1.16 times for D-AF and 1.38 times for T-AF compared to those born in hospitals (ARRR 1.16, CI 1.08, 1.24; ARRR 1.38, CI 1.24, 1.53, respectively). Higher birth order was a common factor, particularly for D-AF, which had 1.22 times (ARRR 1.22, CI 1.17, 1.27) higher risk in third or later-born children. Early pregnancies (under 18) raised the risk of T-AF by 1.28 times (ARRR 1.28, CI 1.12, 1.46), while pregnancy between 18 and 25 increased the risk of S-AF by 1.07 times (ARRR 1.07, CI 1.01, 1.13) compared to those over 25. Lower maternal education levels were linked to a higher prevalence of all forms of AF, particularly for T-AF, which was 1.91 times (ARRR 1.91, CI 1.67, 2.19) more common among uneducated mothers. Inadequate ANC increased D-AF risk by 1.05 times (ARRR 1.05, CI 1.01, 1.09). Underweight mothers exhibited over twice the odds for T-AF (ARRR 2.01, CI 1.88, 2.15), and children of mothers with RMC had 1.10 times (ARRR 1.10, CI 1.07, 1.14) higher risk of S-AF. Anemic mothers increased the risk of D-AF by 1.08 times (ARRR 1.08, CI 1.05, 1.12). Muslim children were at higher risk of S-AF and D-AF being 1.19 times and 1.17 times (ARRR 1.19, CI 1.12, 1.25; ARRR 1.17, CI 1.10, 1.24, respectively). Socially disadvantaged groups (STs, SCs, and OBCs) showed even greater vulnerability, particularly ST children who had 1.79 times (ARRR 1.79, CI 1.59, 2.02) higher risk of T-AF. Lower wealth index was a common risk factor, children from poorer and middle-wealth index families had elevated risks for T-AF, with prevalence rates 1.82 and 1.31 times higher (ARRR 1.82, CI 1.66, 2.00; ARRR 1.31, CI 1.18, 1.46, respectively) (Table 3).

**Table 3:** Factors affecting SAF in rural areas of India based on the latest data (NFHS 5).

Explanatory Factors	Single AF			Double AF			Triple AF		
	ARRR (95% CI)	z	p-value	ARRR (95% CI)	z	p-value	ARRR (95% CI)	z	p-value
Birth Weight (Non-LBW ®)									
LBW	1.32 (1.27, 1.38)	13.01	<0.001	1.85 (1.78, 1.93)	28.55	<0.001	2.33 (2.18, 2.50)	24.42	<0.001
Place of birth (Institution ®)									
Home	1.04 (0.97, 1.11)	1.12	0.264	1.16 (1.08, 1.24)	4.22	<0.001	1.38 (1.24, 1.53)	5.88	<0.001
Birth order number (≤ 2 ®)									
> 2	1.13 (1.09, 1.17)	6.49	<0.001	1.22 (1.17, 1.27)	9.91	<0.001	1.17 (1.09, 1.25)	4.46	<0.001
Mother’s age at 1st childbirth (>25 years ®)									
< 18 years	1.00 (0.93, 1.07)	0.05	0.960	1.05 (0.98, 1.14)	1.39	0.164	1.28 (1.12, 1.46)	3.65	<0.001
18–25 years	1.07 (1.01, 1.13)	2.49	0.013	1.01 (0.95, 1.07)	0.32	0.747	1.02 (0.92, 1.14)	0.41	0.682
Mother’s education (Higher ®)									
No education	1.28 (1.20, 1.36)	7.69	<0.001	1.67 (1.56, 1.80)	14.40	<0.001	1.91 (1.67, 2.19)	9.38	<0.001
Primary	1.19 (1.12, 1.27)	5.21	<0.001	1.44 (1.33, 1.55)	9.58	<0.001	1.74 (1.51, 2.00)	7.67	<0.001
Secondary	1.10 (1.05, 1.16)	3.91	<0.001	1.23 (1.16, 1.31)	6.96	<0.001	1.37 (1.21, 1.54)	5.08	<0.001
Full antenatal care (Yes ®)									
No	0.98 (0.95, 1.01)	−1.28	0.200	1.05 (1.01, 1.09)	2.76	0.006	1.04 (0.98, 1.10)	1.22	0.223
Mother’s BMI (Normal ®)									
Underweight	1.15 (1.11, 1.20)	6.85	<0.001	1.48 (1.42, 1.55)	18.79	<0.001	2.01 (1.88, 2.15)	20.63	<0.001
Overweight/obese	0.76 (0.73, 0.79)	−12.37	<0.001	0.70 (0.67, 0.74)	−13.63	<0.001	0.58 (0.52, 0.64)	−10.00	<0.001
Mother’s WHR (No RMC ®)									
RMC	1.10 (1.07, 1.14)	5.88	<0.001	1.00 (0.97, 1.04)	0.02	0.983	0.98 (0.92, 1.04)	−0.61	0.544
Mother’s anaemia (Non-anaemic ®)									
Anaemic	1.03 (0.99, 1.06)	1.58	0.113	1.08 (1.05, 1.12)	4.48	<0.001	1.06 (0.99, 1.12)	1.77	0.077
Religion (Hindu ®)									
Muslim	1.19 (1.12, 1.25)	6.33	<0.001	1.17 (1.10, 1.24)	5.25	<0.001	1.09 (0.98, 1.22)	1.65	0.099
Christian	0.91 (0.82, 1.02)	−1.60	0.110	0.81 (0.71, 0.91)	−3.44	0.001	0.95 (0.78, 1.17)	−0.45	0.655
Other	0.76 (0.69, 0.84)	−5.21	<0.001	0.74 (0.66, 0.83)	−5.04	<0.001	0.65 (0.52, 0.81)	−3.74	<0.001
Social categories (General ®)									
Scheduled tribe	1.29 (1.21, 1.37)	8.14	<0.001	1.43 (1.34, 1.53)	10.63	<0.001	1.79 (1.59, 2.02)	9.58	<0.001
Scheduled caste	1.29 (1.23, 1.36)	9.87	<0.001	1.41 (1.33, 1.49)	11.73	<0.001	1.54 (1.38, 1.71)	7.75	<0.001
Other Backward Classes	1.20 (1.14, 1.25)	7.82	<0.001	1.23 (1.17, 1.30)	8.11	<0.001	1.41 (1.28, 1.55)	6.78	<0.001
Wealth Index (Rich ®)									
Poor	1.38 (1.32, 1.44)	14.61	<0.001	1.76 (1.67, 1.84)	22.50	<0.001	1.82 (1.66, 2.00)	12.52	<0.001
Middle	1.20 (1.15, 1.26)	7.91	<0.001	1.37 (1.30, 1.44)	11.60	<0.001	1.31 (1.18, 1.46)	5.14	<0.001

**Note:**

• The table aims to identify the factors contributing to the severity of anthropometric failure (AF) in rural areas of India. To achieve this, the researchers used a Multinomial logistic regression (MLR) model because the dependent variable SAF was categorical and had four categories (SAF = No AF, Single AF, Double AF, Triple AF). In the model, “No AF” was used as the reference for the dependent variable, while “®” was used for independent variables.

• The Adjusted Relative Risk Ratio (ARRR) was used to indicate the cumulative odds of the independent factors for each category of the dependent variable relative to “No AF”. The 95% level of confidence interval was used to determine the lower and upper bounds of ARRR. Additionally, the z-value was used to indicate the rate of influence of the independent factors on each category of the dependent variables, while the p-value was used to indicate the significance level for ARRR.

• The study found that low birth weight, birth order, mother’s education, mother’s BMI, social categories, and wealth index are factors that are common to single, double, and triple AF.

## Discussion

This study provides critical insights into the trends and severity of anthropometric failure (SAF) among under-five children in both urban and rural settings in India. While there has been significant progress in child nutrition over the past three decades, our findings illustrate that rural areas continue to exhibit a higher prevalence of AF, particularly in the forms of D-AF and T-AF. The consistency of S-AF over time indicates that despite improvements, critical challenges remain, necessitating targeted interventions.

An inequality was found not only in child nutrition but also in socioeconomic and social determinants between urban and rural populations. Urban residents typically experience more favourable socioeconomic conditions compared to their rural counterparts. This is evident in a higher percentage of families with a higher wealth index (69.11% of rich wealth index), more babies were born in medical institutions, and smaller family units that often limit themselves to two children. Moreover, mothers in urban settings tend to be older when giving birth, possess higher educational qualifications, and receive full antenatal care. Additionally, these mothers typically display lower incidences of underweight conditions and anaemia.

In contrast, rural areas face significant poverty challenges (58.24% of poor wealth index) that impact their populations disproportionately. These areas often have more people from Scheduled Tribes and Castes, which adds to their social and economic challenges. Maternal health in rural regions is concerning, with higher rates of maternal anaemia and underweight mothers, along with lower education than in urban. Such disparities are not only present in India but also reflect common patterns observed in numerous LMICs, underscoring an urgent need for focused efforts to tackle these inequalities [31–34].

A notable risk factor for AF identified in this study was low birth weight. This connects with similar studies in Ethiopia and Tanzania, where low birth weight was linked to stunting [35,36]. This shows how important it is to focus on maternal health during pregnancy, as low birth weight can lead to stunting. The findings were in line with studies conducted in Tanzania and Myanmar and showed that children born at home have a higher risk of developing AF than children born in institutions [36,37]. This disparity likely arises from the fact that institutional births provide better access to skilled obstetric and medical care, highlighting the imperative for increasing institutional deliveries across rural areas.

The relationship between maternal education and child nutrition cannot be overstated. Lower levels of maternal education were associated with higher rates of AF. This finding resonates with previous studies from various nations, including Bangladesh, Yemen, Tanzania, Nairobi, Burkina Faso, and Malawi, indicating that educated mothers are more likely to access vital health information for their children [6,7,38–41].

Maternal education, particularly the quality of education received, is crucial in promoting both child and maternal health. Research consistently shows that mothers with higher levels of education possess a greater understanding of essential health concepts, which directly influences their ability to provide effective antenatal and postnatal care [42]. These educated mothers are typically more informed about important health practices, such as regular medical check-ups during pregnancy, the need for vaccinations, and the significance of proper nutrition for themselves and their children [42–44]. Thus, educational interventions aimed at women could serve as a powerful tool in combating child undernutrition.

Our findings also underscore the significance of antenatal care; women who did not receive such care were at a heightened risk for having children affected by AF. This is consistent with global studies that have documented similar trends, suggesting that access to prenatal healthcare is crucial for improving maternal and child health outcomes in Bangladesh, Peru, Nepal, Yemen, and Thailand [20,45–48]. Notably, mothers with a normal BMI, indicating nutritional well-being are less likely to be undernourished in their children, which have also been observed in Ethiopia [49]. In this regard, it was pointed out poor maternal nutritional status as a risk factor for infant deficit and fetal growth scaling down, and consequently yields to low birth weight [50].

In urban areas, specific risk factors such as maternal anemia and central obesity, as indicated by higher waist-to-hip ratios, have emerged as notable contributors to AF. According to the WHO, a waist circumference greater than hip circumference increases the risk of metabolic complications, indicating central obesity [29]. Moreover, our study highlights a concern regarding specific communities, particularly religious Muslims, Scheduled Tribes, and Scheduled Castes, who are disproportionately affected by AF. The interplay between wealth index and AF prevalence further complicates the issue; indeed, socioeconomic factors play a significant role in child nutrition. Low-income households are often unable to provide adequate nutrition, resulting in poor child health outcomes.

The findings from this study echo trends observed in other LMICs, where low maternal education and income, along with inadequate prenatal care, maternal undernutrition, small birth size markedly affect child nutrition, such as Tanzania, Ethiopia, Bangladesh, Pakistan and sub-Saharan African countries [7,8,13,14,18,20].

To effectively address child undernutrition, India needs to develop multifaceted policies that encompass both urban and rural perspectives. The criteria outlined in Supplementary Table (S2 Table) should serve as a foundational guide for formulating these policies, as they reflect critical characteristics commonly associated with AF in both settings. It will be crucial to prioritize initiatives targeting low birth weight, maternal undernutrition, wealth index, and children from socially vulnerable communities in order to make progress toward the 2030 Agenda for Sustainable Development goals [51].

### Strengths and limitations of the study

The present study examines the nutritional status trends of children from 1992 to 2021 through the analysis of five rounds of NFHS data. The study seeks to address child undernutrition by integrating wasting, stunting and underweight into a common platform with the CIAF concept. The present study also attempted to account for the influence of numerous variables, considering as maternal and socio-demographic characteristics, but they left out certain others associated with genetics, environment, and food. However, additional investigation is required to fully grasp the situation by identifying all potential causes of child malnutrition in India. The data collected through a cross-sectional survey method, however, impedes the establishment of causality in the results. Furthermore, the possibility of social desirability and recall biases among respondents may have influenced the findings, and thus the accuracy of the results.

## Conclusion

The nutritional status of Indian under-five children has improved significantly over the past three decades, with a marked decrease in the prevalence of anthropometric failure from 1992 to 2021. However, currently, more than two-fifths of these children are still affected by either S-AF or D-AF, particularly in rural areas, highlighting an urgent need for targeted interventions.

Childhood anthropometric failure is a multi-faceted phenomenon influenced by various factors, including maternal health and socio-demographic characteristics. To effectively address these challenges, a comprehensive approach is essential. This should involve enhancing maternal health, expanding educational opportunities, and implementing community-based nutrition programs, especially for vulnerable groups such as those from Scheduled Castes and Scheduled Tribes. Moreover, targeted strategies must consider the unique needs of both urban and rural populations, with a focus on reducing low birth weight, improving maternal nutrition, and enhancing the economic conditions of families to combat childhood undernutrition effectively.

## Supporting information

S1 Table Classification of severity of anthropometric failure (SAF) based on CIAF. (DOCX)

S2 Table Top five factors affecting SAF among under-five children in Urban and rural India. (DOCX)

S1 Fig Heatmap showing percentage distribution of SAF across Indian States/Union Territories. (A) Urban area, and (B) Rural area.
(DOCX)

S1 File Data. (SAV)

## Abbreviations

AF: Anthropometric Failure
ANC: Antenatal Care
ARRR: Adjusted Relative Risk Ratio
BMI: Body Mass Index
CI: Confidence Interval
CIAF: Composite Index of Anthropometric Failure
D-AF: Double Anthropometric Failure
DHS: Demographic and Health Surveys
IFA: Iron Folic Acid
IIPS: International Institute for Population Sciences
LBW: Low Birth Weight
LMICs: Low- and Middle-Income Countries
MLR: Multinomial Logistic Regression
N-AF: No Anthropometric Failure
NFHS-5: National Family Health Survey, India 2019-21
OBC: Other Backward Classes
PPI: Patient and Public Involvement
RMC: Risk of Metabolic Complications
SAF: Severity of Anthropometric Failure
S-AF: Single Anthropometric Failure
SC: Scheduled Caste
SDGs: Sustainable Development Goals
ST: Scheduled Tribe
T-AF: Triple Anthropometric Failure
TT: Tetanus Toxoid
UNICEF: United Nations Children’s Fund; Maternal, Newborn, Child and Adolescent Health
VIF: Variance Inflation Factor
WHO: World Health Organization
WHR: Waist-Hip Ratio.
