# Supplementary material for: Assessment of childhood undernutrition in India using National Family Health Surveys: Severity of anthropometric failure and contributing factors
Source: PLoS One. 2026 Feb 11;21(2):e0336335. doi: 10.1371/journal.pone.0336335 (PMC12893611; doi:10.1371/journal.pone.0336335)
Supplement: S1 Table — (DOCX) [file pone.0336335.s001.docx]

| **S1 Table:** Classification of severity of anthropometric failure (SAF) based on CIAF | | | | | |
| --- | --- | --- | --- | --- | --- |
| Group | CIAF | SAF | | | |
|  |  | No AF | Single AF | Double AF | Triple AF |
| A | No failure | Yes | No | No | No |
| B | Wasting only | No | Yes | No | No |
| C | Wasting and Underweight | No | No | Yes | No |
| D | Wasting, Stunting, Underweight | No | No | No | Yes |
| E | Stunting and Underweight | No | No | Yes | No |
| F | Stunting only | No | Yes | No | No |
| Y | Underweight only | No | Yes | No | No |
