# Supplementary material for: Assessment of childhood undernutrition in India using National Family Health Surveys: Severity of anthropometric failure and contributing factors
Source: PLoS One. 2026 Feb 11;21(2):e0336335. doi: 10.1371/journal.pone.0336335 (PMC12893611; doi:10.1371/journal.pone.0336335)
Supplement: S2 Table — (DOCX) [file pone.0336335.s002.docx]

| **S2 Table:** Top five factors affecting SAF among under-five children in Urban and rural India | | | |
| --- | --- | --- | --- |
| Rank | Single AF | Double AF | Triple AF |
|  | ***Urban*** |  |  |
| 1st | Low Birth Weight (z 8.34) | Low Birth Weight (z 16.76) | Low Birth Weight (z 12.84) |
| 2nd | Religious Muslims (z 7.51) | Poor wealth index (z 10.42) | Secondary education (z 7.25) |
| 3rd | Secondary education (z 5.15) | No education (z 8.92) | Primary education (z 6.93) |
| 4th | Scheduled tribe (z 4.21) | Underweight mother (z 8.73) | Underweight mother (z 6.41) |
| 5th | No education (z 4.16) | >2 birth order (z 8.64) | Poor wealth index (z 6.11) |
|  |  |  |  |
|  | ***Rural*** |  |  |
| 1st | Poor wealth index (z 14.61) | Low Birth Weight (z 28.55) | Low Birth Weight (z 24.42) |
| 2nd | Low Birth Weight (z 13.01) | Poor wealth index (z 22.50) | Underweight mother (z 20.63) |
| 3rd | Scheduled caste (z 9.87) | Underweight mother (z 18.79) | Poor wealth index (z 12.52) |
| 4th | Scheduled tribe (z 8.14) | No education (z 14.40) | Scheduled tribe (z 9.58) |
| 5th | Middle wealth index (z 7.91) | Scheduled caste (z 11.73) | No education (z 9.38) |
| *Education - stands for mothers institutional education* | | | |
