# Supplementary material for: Assessment of childhood undernutrition in India using National Family Health Surveys: Severity of anthropometric failure and contributing factors
Source: PLoS One. 2026 Feb 11;21(2):e0336335. doi: 10.1371/journal.pone.0336335 (PMC12893611; doi:10.1371/journal.pone.0336335)
Supplement: S1 Fig — (A) Urban area, and (B) Rural area. (DOCX) [file pone.0336335.s003.docx]

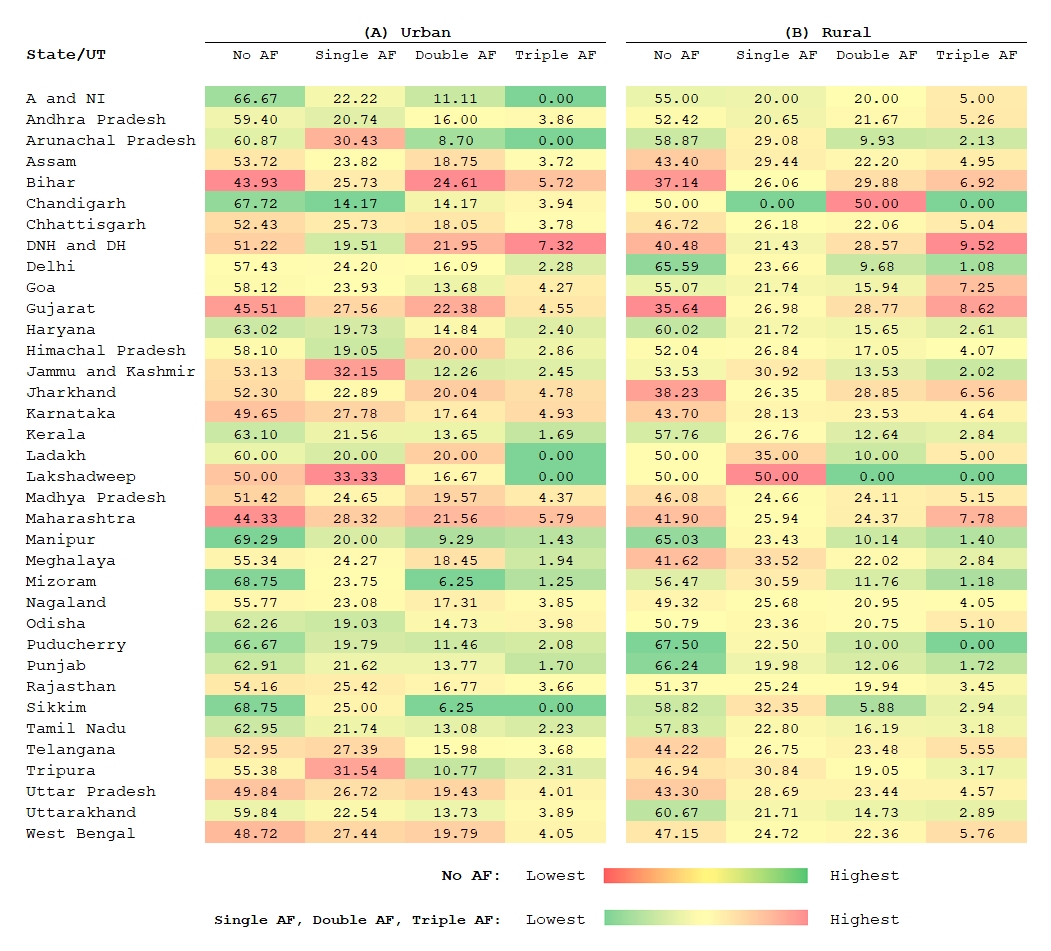


*Note:*

*Comparisons of percentages among States/Union Territories are displayed using cell colour;*

*A and NI: Andaman and Nicobar Islands; DNH and DH: Dadra & Nagar Haveli and Daman & Diu;*

*J and K: Jammu and Kashmir UT: Union territory*

**S1Fig:** Heatmap showing percentage distribution of SAF across Indian States/Union Territories,

(A) Urban area, and (B) Rural area
